# Avoiding Emotional Bonds: An Examination of the Dimensions of Therapeutic Alliance Among Cannabis Users

**DOI:** 10.3389/fpsyt.2013.00070

**Published:** 2013-07-22

**Authors:** Alison Healey, Frances Kay-Lambkin, Jenny Bowman, Steven Childs

**Affiliations:** ^1^School of Psychology, University of Newcastle, Callaghan, NSW, Australia; ^2^Central Coast Drug and Alcohol Clinical Service, Northern Sydney Central Coast Area Health Service, Gosford, NSW, Australia; ^3^National Drug and Alcohol Research Centre, University of New South Wales, Sydney, NSW, Australia; ^4^Centre for Translational Neuroscience and Mental Health, Faculty of Health, University of Newcastle, Callaghan, NSW, Australia

**Keywords:** **c**annabis use, therapeutic alliance, treatment engagement, substance misuse, Internet

## Abstract

There is a growing need to provide treatment for cannabis users, yet engaging and maintaining this population in treatment is particularly difficult. Although past research has focused on the importance of therapeutic alliance on drug treatment outcomes, this is the first study to examine the dimensions of therapeutic alliance for cannabis users compared with users of alcohol or other drugs in a naturalistic setting. The acceptability of Internet-delivered interventions for drug and alcohol treatments is also investigated. Participants (*n* = 77) included clients who were receiving outpatient drug and alcohol treatment at a publicly funded health service, including a Specialist Cannabis Clinic. The results indicated that one particular domain of alliance, Bond, was consistently lower, from both client and clinician perspectives, for current cannabis users relative to those not currently using cannabis. Client perceptions of Bond decreased as the severity of cannabis use increased (*r* = −0.373, *p* = 0.02). Cannabis Clinic clients did not report a significantly lower Bond with their clinicians, suggesting that specialized cannabis services may be better placed to provide appropriate treatment for this population than embedding cannabis treatment within traditional drug and alcohol treatment teams. In addition, Internet/computer-based treatments may be one potential way to engage, transition, or retain cannabis users in treatment.

## Introduction

Cannabis is the most common illicit substance in the US, Australia, and in most developed countries, and is increasing in popularity (1, 2). However, fewer than 10% of cannabis users access treatment for their cannabis use directly (3).

For people with cannabis use problems (as with users of other substances), the effects of the drug itself, multiple co-morbidities, and/or chaotic lifestyles present constant challenges to treatment engagement, often resulting in short windows of opportunity in which to provide treatment (4). However, when cannabis users do attend counseling treatments, including computerized therapy, they report significant improvements in mood and substance use (5).

There is therefore a need for research to target cannabis users in treatment, and to better understand and identify potential strategies to maximize engagement. This is particularly important given the evidence suggesting cannabis users respond better with longer term treatment (5, 6), and that therapeutic alliance may be an important factor in maintaining cannabis users in treatment over the longer term (8, 9).

### Therapeutic alliance

It is generally accepted that forming a strong therapeutic alliance during counseling will improve treatment outcomes (7, 8). Therapeutic alliance is multidimensional, but generally refers to the nature of the affective bond and collaborative relationship between the client and therapist, who are working in cohesion on therapeutic goals and tasks (9, 10). The therapeutic bond is considered a core component of alliance, and is assessed in most therapeutic alliance measures. Bond encompasses the emotional connection, understanding, and support in the client/therapist relationship. Other dimensions of alliance, such as tasks, goals, and partnership, are considered more intellectual and outcome-based, focusing on the client and therapist working jointly toward therapeutic goals (11, 12).

### The effect of cannabis use on alliance and treatment outcomes

Only one previous study has reported on the relationship between therapeutic alliance and cannabis use. Diamond et al. (13) conducted an investigation using the Cannabis Youth Treatment Study population (*n* = 600) to explore the impact of therapeutic alliance on treatment outcomes and attendance in adolescent cannabis users. Early alliance, as perceived by the clients, predicted fewer days of cannabis use at both 3 and 6 months follow-ups.

A range of factors is suggested to have an impact on the establishment of therapeutic alliance, regardless of the clinical group under consideration. These include mental health severity (in particular symptoms of depression and anxiety), age, and gender (14) and should be considered in any examination of alliance in treatment. However, other factors, unique to substance using populations and cannabis users in particular, may pose further threats to the establishment of therapeutic alliance in this group. For example, the intoxication and long-term physiological effects of cannabis may in part explain why this group may be challenging to engage and retain in traditional substance abuse counseling treatment. Euphoria, relaxation, time distortion, perceptual alterations, intensified sensory experience, loss of sense of personal identity, difficulties with the formation and retrieval of memories and attention difficulties are associated with the short-term effects of cannabis use (15, 16). Cannabis use also has psychological impacts on anxiety, panic, depression, and psychosis, which can be associated with chronic consumption (17). In addition, research findings by Kay-Lambkin et al. (18) suggested that people who used cannabis presented to a treatment trial with significantly lower levels of functioning [*F*(1, 223) = 6.009, *p* = 0.015], and significantly higher levels of interpersonal sensitivity [*F*(1, 216) = 4.674, *p* = 0.032], than did users of other drugs. Interpersonal sensitivity was indicative of recent distress due to feelings of self-consciousness and inferiority, having feelings easily hurt, and others being unfriendly or disapproving. Cannabis users also reported significantly higher paranoid ideation than did users of other drugs [*F*(1, 218) = 9.042, *p* = 0.003]. These factors are suggestive of the potential difficulties for cannabis users to engage in therapy and could potentially result in them feeling disconnected from the treatment process and their therapist, impacting on alliance. The may be particularly true for the emotional aspects of therapeutic alliance, namely the core component of therapeutic bond.

### The integration of Internet-delivered treatments for drug and alcohol clients

Internet-based treatments may be one-way of improving engagement with cannabis users in a treatment for their cannabis use, given their heightened paranoia, anxiety, and interpersonal sensitivity relative to users of other drugs. Such treatments have been shown to be effective across a number of therapeutic contexts (19), and as the demand for Drug and Alcohol Services has remained strong, the use of Internet-based treatments has grown into the general substance abuse field (20). After comparing Internet-based interventions with traditional psychotherapy, and finding similar results between the two methods, Carroll et al. (21) provide support for the idea of a computer-assisted therapist model to augment the treatment provided by clinicians working in services as a feasible way of improving treatment outcomes. Marsch (22) also supports this idea, claiming that technology-based interventions for substance use disorders can function as “clinician-extenders” to reduce some of the barriers to treatment.

In the first RCT conducted in this area, Kay-Lambkin et al. (5) investigated the clinical efficacy of the SHADE (Self-Help for Alcohol and other drug use and Depression) computer treatment program. It consisted of 10 sessions of combination motivational interviewing and cognitive behavior therapy delivered via a computer program, and was compared to a therapist-delivered equivalent. Results indicated that both computer- and therapist-delivered treatments yielded similar outcomes in substance use and depression at 12-month follow-up. Interestingly, SHADE was most efficacious for people with cannabis use problems and co-morbid depression when compared to other substances of concern and therapist-delivered treatment, with SHADE cannabis users reporting twice the reductions in cannabis use as their therapist-delivered counterparts (5).

### The current study

This study aims to investigate the association between cannabis use and therapeutic relationship within a publicly funded Drug and Alcohol Service, from both client and clinician perspectives, comparing a general counseling and cannabis-specific services. It is hypothesized that current users of cannabis will report lower levels of therapeutic alliance relative to people not currently using cannabis. It will also examine the acceptability of computers/the Internet in providing information about and treatment for alcohol/other drug use, hypothesizing that cannabis users will be more open to these modalities than users of other drugs.

## Materials and Methods

### Participants

A total of 77 client participants who were receiving outpatient drug and alcohol treatment at the Northern Sydney Central Coast Area Health Service (NSCCAHS, NSW, Australia) were recruited to this study. Participants were referred from the teams within the counseling portfolio including; Cannabis Clinic (*n* = 21), Drug and Alcohol Counseling (*n* = 50), and The Magistrates Early Referral into Treatment program (MERIT) (*n* = 6). Participants were aged between 19 and 69, with a mean age of 40. The majority of participants were Australian born (*n* = 69, 89.6%), with 2.6% (*n* = 2) of Aboriginal or Torres Strait Islander decent. One third of participants were living alone (*n* = 25), 31% were living with a spouse and/or children (*n* = 24) and 14% were living with their parents (*n* = 11). Sixty-four percent were unemployed (*n* = 49), 55% were males (*n* = 42) and 46% were single and had never married (*n* = 35). At baseline assessment, 51% (*n* = 39) participants indicated they were seeking treatment primarily for concerns regarding alcohol, 27% (*n* = 21) for cannabis, 9% (*n* = 7) for methamphetamine, 6% (*n* = 5) for tobacco, and 3% (*n* = 2) for heroin and hallucinogens (ecstasy) respectively. Abuse/dependence criteria were not measured as part of the current study, however information provided by the referring clinicians indicated that the majority of the sample (*n* = 56, 72%, those referred via the MERIT and Drug and Alcohol Counseling programs) met DSM-IV criteria for alcohol dependence at study entry. Twenty-one participants (27%, those referred via the Cannabis Clinic) met criteria for cannabis dependence. Participants could have met dependence criteria for several substances concurrently, however all participants with cannabis dependence were referred via the Cannabis Clinic. No participants referred to this service were excluded from participation in the study.

The clinician participants in this study (*n* = 16) were employes of NSCCAHS with tertiary qualifications in psychology (*n* = 5) or nursing (*n* = 11) and were registered in their fields with the relevant professional organization. Participants in the clinician group reported a mean age of 42.90 years (SD = 11.17, Range 25–58) and were, for the most part, female (*n* = 11/16). They all provided assessment and treatment according to evidence-based psychosocial guidelines (23).

### Measures

A range of demographic and treatment variables were assessed at the baseline interview. These included age, gender and marital status as per Kay-Lambkin et al. (24).

The Opiate Treatment Index [OTI; (25)] was used to assess the quantity and frequency of alcohol/other drug use across 11 individual substances (alcohol, cannabis, heroin, other opiates, methamphetamine, cocaine, tranquilizers, barbiturates, hallucinogens, inhalants, tobacco). For each substance, clients were asked to report on their last three use occasions in the month prior to assessment, estimating the amount of each drug consumed on each of these occasions. An average use index for the previous month was calculated (OTI *q* score) as an estimate of quantity and frequency of use, with a score of 1 indicating once daily use, 2 twice daily use, and so on. Participants received an OTI *q* score for each substance. Cannabis use (OTI *q* score – Cannabis) was used as a continuous variable in the analysis, and was also categorized according to the following to facilitate comparisons with users of other drugs combined (including alcohol):

“Current cannabis use” – this group included people who reported using any level of cannabis at baseline (*n* = 37) and was compared to people not using any cannabis currently (but were using other drugs including alcohol), as measured by the OTI *q* score (*n* = 40). A proportion of “current cannabis use” participants (*n* = 21, referred via the Cannabis Clinic) met criteria for cannabis dependence, however the remainder (*n* = 16) did not. This provided a group reporting a range of severity of cannabis use to be analyzed. No participants in the comparison group met criteria for cannabis dependence at study entry. All participants, regardless of categorization, could have been using other substances concurrently.
“Cannabis Clinic” – this group included people referred to the study by the Cannabis Clinic clinicians (*n* = 21) and was compared to people seeking treatment from the Drug and Alcohol Counseling and MERIT teams (*n* = 56). The Cannabis Clinic comprised clinicians specifically trained in engaging and treating clients with cannabis use problems, and offers services to people aged 16 years and over who want to quit or reduce their cannabis use (23). All Cannabis Clinic clients met criteria DSM-IV criteria for cannabis dependence (provided by the referring clinician). The comparison group were using a range of substances, but all met criteria for alcohol dependence at study entry.

The DASS-21 (Depression, Anxiety, and Stress Scale) was used to measure depression, anxiety, and stress scores (26). Henry and Crawford (27) assessed the reliability of the DASS-21. A Cronbach alpha of 0.93 for the total measure was found, as well as high reliability for the stress and depression scales and adequate for the anxiety scale (0.93, 0.90, and 0.82 respectively).

The Agnew Relationship Measure (ARM) Client Questionnaire (11) was used to measure therapeutic alliance from both client and clinician perspectives. The dimensions include Bond (acceptance, support, and understanding in the relationship), Partnership (working together of tasks in therapy), Confidence (respect and optimism for the therapist’s competence), Openness (client’s feeling toward disclosure of personal information without embarrassment or fear), and Client Initiative (client’s responsibility for direction in therapy). It consists of 28 items used to measure the five dimensions of the client-therapist alliance. Agnew-Davis et al. (11) rated the internal consistency and found that all dimensions had an alpha value between 0.77 and 0.87, except for Client Initiative, which was 0.55. The ARM has been used in prior drug and alcohol research and also has strong convergent validity with the Working Alliance Inventory [WAI; (28)] another popular method of measuring alliance (12).

The Computer Anxiety Questionnaire which includes a Computer Anxiety rating scale [CARS; (29)] and a Computer Thoughts Survey [CTS; (29)] were used to measure client’s opinions and anxiety around using computers. The CARS asks respondents to indicate on a Likert scale (“very much” to “not at all”) how anxious each of 20 items would make them feel. The CTS asks respondents to indicate how often they currently have one of 20 specified thoughts when they use a computer or think about using a computer (“not at all” to “very much”).

Further questions were also asked to explore clients’ openness to using computer/Internet-delivered treatment for their primary drug of concern. The questions included; “*Have you ever used any computer or Internet-delivered treatment for your mental health or drug and alcohol use issues*?” and “*If you were offered computer/Internet-delivered treatment*, *would you utilize it as part of your treatment for drug and alcohol and/or mental health problems*?” Using a 4-point Likert scale from “not at all” through to “a lot” clients were also asked to rate how much they agreed with the following statements; “*Computer*/*Internet-delivered treatment could be useful in helping me deal with my alcohol and other drug use*” and “*Computer*/*Internet-delivered treatment could be useful in helping me find information about alcohol/other drug use*.” The Likert scale questions were then re-coded into two categories (not at all and not much = no, a little and a lot = yes) due to sample size and for ease of analysis.

### Research design

The detailed methods and study design have been outlined elsewhere (24). The study received ethics approval from the relevant organizations (e.g., Northern Sydney Central Coast Human Research Ethics Committee Approval Number: 08/HARBR/78/79).

Participating clinicians from the Cannabis Clinic and MERIT teams were asked to introduce the research project and gain consent to release their client’s contact details to the research team during the initial assessment. It was necessary to ensure that the clients’ decision to participate in the research was entirely independent of their treatment with the service, and that if they did not choose to participant their treatment would not be impacted on in anyway. There was a longer waitlist for clients seeking treatment from the Counseling Team therefore consent to release contact details was done while clients were on the waitlist in order to engage them with the research earlier in their treatment episode.

Clients were contacted via phone by the research assistant and invited to participate in the study. At this stage clients were asked if they agreed to be contacted in 1 week, during which they received and considered the information sheet and consent form for the study. Following the provision of informed consent, assessment measures were collected over the phone, Clients were offered a $20 reimbursement for their time for completing the phone assessments. Clinicians were unaware if the clients they had referred had consented to the research, unless told so by the client in therapy. Clinicians completed therapeutic alliance measure for all clients after the initial session for the duration of the study.

## Results

### Recruitment

Recruitment for this study was from August 2010 through to April 2011. During this time 166 clients were referred, of which 56 refused participate, 24 were unable to be contacted and 9 did not return their consent documents.

### Statistics and data analysis

Chi-square analysis was used to examine the interaction between the two variables “Current cannabis use” (yes/no) and “Cannabis Clinic” (yes/no; $X_{1}^{2}=28.14$, *p* = 0.00, *n* = 77). All participants referred from the cannabis clinic (*n* = 21, 27% of total) reported cannabis use at baseline. Of the remainder (those referred from the Counseling/MERIT teams), 18 (23% of total) reported current cannabis use, and 38 (49%) reported no current cannabis use. No participant referred from the Cannabis Clinic reported zero cannabis use at baseline. Given the significant overlap between the “Current cannabis use” and “Cannabis Clinic” variables, and the zero value of the cell Cannabis Clinic + no baseline cannabis use, separate one-way Analysis of Variance (ANOVA) are reported for examining relationships between each of the cannabis variables (Current cannabis use and Cannabis Clinic) and the outcome of interest (e.g., therapeutic alliance, depression, anxiety, stress), rather than conducting a two-way ANOVA.

At baseline, participants reported use of a range of substances in the month prior to assessment, including alcohol (Mean = 3.74, SD 5.62 standard drinks per day), heroin (Mean = 0.10, SD 0.31 use occasions per day), cannabis (Mean = 3.86, SD 8.18 use occasions per day), other opiates (Mean = 0.22, SD 0.62 use occasions per day), methamphetamine (Mean = 0.16, SD 0.45 use occasions per day), cocaine (Mean = 0.19, SD 0.61 use occasions per day), tranquilizers (Mean = 0.23, SD 0.62 use occasions per day), barbiturates (Mean = 0.16, SD 0.49 use occasions per day), hallucinogens (Mean = 0.16, SD 0.49 use occasions per day), inhalants (Mean = 0.21, SD 0.62 use occasions per day), and tobacco (Mean = 12.28, SD 12.64 cigarets per day). This was based on participant self-report using the OTI, with a score of 0.14 equating to once weekly use for the prior month, a score of 1 indicating once daily use for the prior month, and so on. Polydrug use was common, with participants reporting an average use of 2–3 drug types in the month prior to baseline (including tobacco; Mean = 2.60, SD 1.55). One-way ANOVAs indicated no significant differences in the use of any drug type (with the exception of cannabis) at baseline for Current versus Non-current cannabis users and participants referred from the Cannabis Clinic versus those who were not. As expected, significantly higher cannabis use was reported by participants referred from the Cannabis Clinic (Mean = 9.39, SD12.17 use occasions per day) versus those referred from the other treatment teams (Mean = 1.50, SD 3.97 use occasions per day; *F*(1, 56 = 13.654, *p* = 0.001). The same was true for Current versus non-current cannabis users.

#### The impact of demographic variables on client perceptions of therapeutic alliance

A one-way ANOVA revealed that there were no significant differences between age and gender on the therapeutic alliance subscales. The youngest age group (19–30) did score lower, on average, on all five dimensions of the therapeutic alliance than the other age groups, however this was not statistically significant. Overall, there was a trend on the openness dimension of alliance related to age [*F*(3, 63) = 2.34, *p* = 0.082], with Bonferroni *post hoc* analysis indicating that 19–30 year olds scored lower than the other age groups. For gender, males on average scored lower across all of the therapeutic alliance dimensions than females, although these differences were not significant.

#### The impact of cannabis use on client perceptions of therapeutic alliance

Correlational analysis using cannabis as a continuous variable (OTI *q* score) was conducted, and revealed a significant negative correlation between the amount of cannabis used and Bond on the client ARM (*r* = −0.373, *p* = 0.02, see Table 1). There were no significant correlations found between amount of cannabis use and any other dimension of the client therapeutic alliance measure.

**Table 1:** **Pearson correlation analysis for depression, anxiety, stress scores (DASS-21), subscales of the Agnew-Davies Relationship Measure (ARM) of therapeutic alliance (client-rated) and past-month cannabis use (OTI *q* Score)**.

	DASS-21 depression	DASS-21 anxiety	DASS-21 stress	Past-month cannabis use^#^
ARM-bond	−0.020	−0.006	0.040	−0.373*
ARM-partnership	0.084	0.118	0.057	−0.196
ARM-confidence	−0.062	−0.114	−0.096	−0.123
ARM-openness	0.103	0.031	0.100	−0.032
ARM-initiative	−0.078	0.069	−0.028	−0.149
DASS-21 depression	###	0.748*	0.883*	−0.063
DASS-21 anxiety	###	###	0.730*	−0.059
DASS-21 stress	###	###	###	−0.078

*p < 0.05 ^#^OTI *q* score.

One-way ANOVAs compared past-month cannabis users with those who did not use cannabis in the past month on the dimensions of the client ARM. There was a significant difference found between people using cannabis in the past month and people who did not on the Bond dimension of the ARM, *F*(1, 65) = 4.923, *p* = 0.03. This result, shown in Table 2, suggests that clients who use any level of cannabis found it significantly more difficult to develop a therapeutic bond with their clinician at the beginning of treatment than those who did not use cannabis.

**Table 2:** **Current cannabis^#^ users compared to no current cannabis users on client-rated subscales of the Agnew-Davies Relationship Measure of therapeutic alliance**.

Therapeutic alliance subscales	N*	Mean	SD	ANOVA
Bond
Current cannabis use	33	5.91	0.821	*F*(1, 65) = 4.923, *p* = 0.03
No current cannabis use	34	6.31	0.674	
Partnership
Current cannabis use	33	5.97	0.848	*F*(1, 64) = 0.511, *p* = 0.477
No current cannabis use	33	6.13	0.929	
Confidence
Current cannabis use	33	5.81	0.842	*F*(1, 65) = 0.853, *p* = 0.359
No current cannabis use	34	6.19	2.17	
Openness
Current cannabis use	33	5.21	0.996	*F*(1, 65) = 0.040, *p* = 0.843
No current cannabis use	34	5.16	1.18	
Initiative
Current cannabis use	33	3.38	0.974	*F*(1, 65) = 0.095, *p* = 0.759
No current cannabis use	34	3.46	1.05	

*^#^Current cannabis use refers to people who nominated they had used cannabis (at any level) in the month prior to assessment. “No current cannabis use” includes people using a range of substance, but no cannabis, in the month prior to assessment*.

* Note some missing data due to incomplete ARM at baseline.

The “Cannabis Clinic” group were compared to people engaged with either the counseling or MERIT teams on the client ARM (see Table 3). There were no significant differences between these groups on the measures.

**Table 3:** **“Cannabis Clinic” group compared to other teams (Counseling and MERIT)^#^ on the client-rated subscales of the Agnew-Davies Relationship Measure of therapeutic alliance**.

Therapeutic alliance subscales	*N*	Mean	SD	ANOVA
Bond
Cannabis clinic	19	5.92	0.73	*F*(1, 65) = 1.664, *p* = 0.202
Counseling/MERIT	48	6.10	0.78	
Partnership
Cannabis clinic	19	5.96	0.94	*F*(1, 64)0.272 = , *p* = 0.604
Counseling/MERIT	47	6.09	0.87	
Confidence
Cannabis clinic	19	6.53	2.79	*F*(1, 65) = 2.758, *p* = 0.102
Counseling/MERIT	48	5.79	0.84	
Openness
Cannabis clinic	19	5.51	1.08	*F*(1, 65) = 2.474, *p* = 0.121
Counseling/MERIT	48	5.06	1.07	
Initiative
Cannabis clinic	19	3.28	1.17	*F*(1, 65) = 0.555, *p* = 0.459
Counseling/MERIT	48	3.48	0.91	

*^#^Cannabis Clinic participants all met criteria for cannabis dependence at study entry, and were referred via a treatment team specifically trained to engage and treat clients with cannabis use problems. Participants from Counseling/MERIT teams did met criteria for alcohol dependence, but not cannabis dependence, at study entry. Participants in each group could have met dependence criteria for other substances concurrently*.

#### The impact of mental health symptoms on therapeutic alliance

There were no significant correlations found between depression, anxiety, and stress (DASS-21) scores and cannabis use in the month prior to survey (OTI *q* scores, see Table 1). In addition, no significant correlations were found between DASS-21 scores and subscales of the ARM.

A one-way ANOVA also indicated no significant difference between current cannabis users and non-cannabis-users on the DASS-21 subscales. However, the mean scores for depression, anxiety, and stress were lower for the “current cannabis” and “Cannabis Clinic” groups, relative to their counterparts (see Tables 4 and 5, respectively).

**Table 4:** Current cannabis* users versus non-current users as a function of current depression, anxiety, and stress, as measured by the DASS-21.

DASS-21 subscales	*N*	Mean	SD	ANOVA
Depression
Current cannabis use	37	16.81	11.99	*F*(1, 75) = 0.194, *p* = 0.661
No current cannabis use	40	18.10	13.57	
Anxiety
Current cannabis use	37	9.84	10.77	*F*(1, 75) = 0.355, *p* = 0.553
No current cannabis use	40	11.25	10.02	
Stress
Current cannabis use	37	19.96	10.52	*F*(1, 75) = 0.869, *p* = 0.354
No current cannabis use	40	22.35	11.84	

*Current cannabis use refers to people who nominated they had used cannabis (at any level) in the month prior to assessment. “No current cannabis use” includes people using a range of substance, but no cannabis, in the month prior to assessment.

**Table 5:** **A comparison of Cannabis Clinic versus MERIT/Counseling Team^#^ participants on measures of depression, anxiety, and stress (DASS-21)**.

DASS-21 subscales	*N*	Mean	SD	ANOVA
Depression
Cannabis clinic	21	15.24	9.02	*F*(1, 75) = 0.890, *p* = 0.349
Counseling/MERIT	56	18.32	13.89	
Anxiety
Cannabis clinic	21	8.10	7.76	*F*(1, 75) = 1.669, *p* = 0.200
Counseling/MERIT	56	11.50	11.08	
Stress
Cannabis clinic	21	18.51	9.50	*F*(1, 75) = 1.685, *p* = 0.198
Counseling/MERIT	56	22.21	11.72	

*^#^Cannabis Clinic participants all met criteria for cannabis dependence at study entry, and were referred via a treatment team specifically trained to engage and treat clients with cannabis use problems. Participants from Counseling/MERIT teams did met criteria for alcohol dependence, but not cannabis dependence, at study entry. Participants in each group could have met dependence criteria for other substances concurrently*.

#### The prediction of client perceptions of therapeutic alliance

Given the associations between cannabis use and the ARM subscale of Bond, a linear regression model was also used to determine the independent contribution of cannabis use to this ARM subscale. Predictor variables were included in the model if their univariate significance was<0.1. Based on this criterion, age, gender, and OTI cannabis *q* scores were included in the regression model. The regression equation was statistically significant in predicting Bond [*F*(3, 63) = 3.800, *p* = 0.014, $r_{\text{adj}}^{2}=0.113$], with cannabis use being the sole significant predictor (*p* = 0.008).

#### The impact of cannabis use on clinician perceptions of therapeutic alliance

One-way ANOVAs were also used to compare the clinician ratings of the therapeutic alliance dimensions of the ARM for cannabis users. As shown in Table 6, there was a significant difference found between current and non-current cannabis users on the Bond subscale of therapeutic alliance [*F*(1, 64) = 4.257, *p* = 0.043], indicating that clinicians seeing clients who were current cannabis users reported a lower therapeutic bond early in therapy than non-current cannabis users.

**Table 6:** Clinician-rated therapeutic alliance (as measured by the Agnew-Davies Relationship Measure) for current versus non-current cannabis users*.

Therapeutic alliance subscales	*N*^#^	Mean	SD	ANOVA
Bond
Current cannabis use	32	5.75	1.01	*F*(1, 64) = 4.257, *p* = 0.043
No current cannabis use	34	6.19	0.70	
Partnership
Current cannabis use	32	6.20	4.50	*F*(1, 64) = 0.098, *p* = 0.756
No current cannabis use	34	6.53	4.12	
Confidence
Current cannabis use	32	5.26	0.99	*F*(1, 64) = 0.740, *p* = 0.393
No current cannabis use	34	5.45	0.80	
Openness
Current cannabis use	32	5.28	4.53	*F*(1, 64) = 0.260, *p* = 0.612
No current cannabis use	34	4.87	1.06	
Initiative
Current cannabis use	32	4.40	1.09	*F*(1, 64) = 0.046, *p* = 0.831
No current cannabis use	34	4.34	1.09	

*Current cannabis use refers to people who nominated they had used cannabis (at any level) in the month prior to assessment. “No current cannabis use” includes people using a range of substance, but no cannabis, in the month prior to assessment.

*^#^Note some missing data due to incomplete ARM at baseline*.

There was also a significant difference found for clinician-rated Bond for the “Cannabis Clinic” participants when compared to those from other teams [*F*(1, 64) = 5.560, *p* = 0.02]. This suggested that Cannabis Clinic clinicians reported lower therapeutic bond with their clients early in therapy, see Table 7.

**Table 7:** **“Cannabis Clinic” group compared to other teams (Counseling and MERIT)^#^ on the clinician-rated subscales of the Agnew-Davies Relationship Measure of therapeutic alliance**.

Therapeutic alliance subscales	N*	Mean	SD	ANOVA
Bond
Cannabis clinic	18	5.57	0.98	*F*(1, 64) = 5.560, *p* = 0.021
Counseling/MERIT	48	6.13	0.80	
Partnership
Cannabis clinic	18	5.22	1.18	*F*(1, 64) 1.790 = , *p* = 0.186
Counseling/MERIT	47	6.80	4.91	
Confidence
Cannabis clinic	18	5.05	1.03	*F*(1, 64) = 2.955, *p* = 0.090
Counseling/MERIT	48	5.47	0.82	
Openness
Cannabis clinic	18	5.82	5.88	*F*(1, 64) = 1.340, *p* = 0.251
Counseling/MERIT	48	4.79	1.27	
Initiative
Cannabis clinic	18	4.11	0.88	*F*(1, 64) = 1.439, *p* = 0.235
Counseling/MERIT	48	4.46	1.14	

*^#^Cannabis Clinic participants all met criteria for cannabis dependence at study entry, and were referred via a treatment team specifically trained to engage and treat clients with cannabis use problems. Participants from Counseling/MERIT teams did met criteria for alcohol dependence, but not cannabis dependence, at study entry. Participants in each group could have met dependence criteria for other substances concurrently*.

*Note some missing data due to incomplete ARM at baseline.

No significant differences were found for any other subscale of the clinician-rated therapeutic alliance between Cannabis Clinic participants and their Counseling/MERIT counterparts. However, a non-significant trend emerged for clinicians who see clients at the Cannabis Clinic to report lower confidence in therapy at baseline compared to clinicians from other teams [*F*(1, 64) = 2.955, *p* = 0.090], see Table 7.

#### Openness to receiving computer-based treatments

Results on the CARS, the CTS, and questions exploring participants’ openness to using a computer-delivered treatment were compared between cannabis and non-cannabis groups using a one-way ANOVA (see Table 8). There were no significant differences found between the cannabis and non-cannabis groups on the CARS and CTS. There was a tendency for the cannabis users to report lower average scores on the CARS than people using other drugs, particularly for referrals from the Cannabis Clinic (*M* = 28.29) compared to other teams (*M* = 31.70). This indicates that cannabis users were somewhat less anxious about the idea of using a computer, albeit that this was not a statistically significant difference. Conversely, current cannabis users and Cannabis Clinic participants reported fewer positive thoughts about computers, although again, this was not statistically significant.

**Table 8:** Responses to the Computer Anxiety Rating Scale (CARS) and the Computer Thoughts Survey (CTS) as a function of current/non-current cannabis* use and Cannabis Clinic versus Counseling/MERIT participants^#^.

	CARS	CTS	
	*Mean (SD)*	Positive thoughts *Mean (SD)*	Negative thoughts *Mean (SD)*
Current cannabis use	29.03 (14.01)	22.43 (9.02)	17.59 (9.13)
No current cannabis use	31.70 (12.56)	24.86 (9.03)	19.62 (7.47)
ANOVA	*F*(1, 75) = 0.78, *p* = 0.38	*F*(1, 72) = 1.35, *p* = 0.25	*F*(1, 72) = 1.09, *p* = 0.30
Cannabis clinic	28.29, (11.63)	22.81, (8.86)	19.48, (10.88)
Counseling/MERIT	31.21, (13.83)	23.98, (9.18)	18.26, (7.20)
ANOVA	*F*(1, 75) = 0.74, *p* = 0.40	*F*(1, 72) = 0.25, *p* = 0.62	*F*(1, 72) = 0.31, *p* = 0.58

*Current cannabis use refers to people who nominated they had used cannabis (at any level) in the month prior to assessment. “No current cannabis use” includes people using a range of substance, but no cannabis, in the month prior to assessment.

*^#^Cannabis Clinic participants all met criteria for cannabis dependence at study entry, and were referred via a treatment team specifically trained to engage and treat clients with cannabis use problems. Participants from Counseling/MERIT teams did met criteria for alcohol dependence, but not cannabis dependence, at study entry. Participants in each group could have met dependence criteria for other substances concurrently*.

A continuity-corrected chi squared analysis was used to explore differences between participants on their openness to using an Internet-delivered treatment for their primary substance of concern. Although there were no significant differences observed, a higher proportion of current cannabis users reported previous use of Internet-delivered treatments compared to non-current cannabis users [53 versus 35%; χ^2^(1) = 0.634, *p* = 0.426]. Similarly, 62% of people from the “Cannabis Clinic” group reported prior use of Internet-delivered treatments compared to 32% from other teams [χ^2^(1) = 1.97, *p* = 0.161]. There was a tendency for cannabis users to think that Internet-delivered treatments could be useful in locating information about or treatment for their primary drug of concern. For example, 87% of current cannabis users people agreed that the Internet would useful in helping them deal with their primary drug, compared to 70% of people with no current cannabis use [χ^2^(1) = 0.675, *p* = 0.411]. Ninety-two percent of Cannabis Clinic participants indicated they would utilize Internet-delivered treatment if offered to them, compared with 75% of people referred from other teams [χ^2^(1) = 0.712, *p* = 0.389].

## Discussion

This is the first study to explore differences in therapeutic alliance for cannabis users compared with users of alcohol or other drugs in a naturalistic setting. The results indicate that one particular domain of alliance, Bond, is consistently different, from both client and clinician perspectives, for current cannabis users relative to people not currently using cannabis. There was evidence for the client’s perception of Bond to decrease as the severity of cannabis use increased. Cannabis use remained a significant independent predictor of client-related Bond when age and gender were taken into account via the regression analysis. This was also the case for clinicians’ perceptions of Bond. The results are elaborated on below.

### The Bond dimension of therapeutic alliance

The Bond dimension is different from the other therapeutic alliance dimensions. Questions on the Bond dimension of the client ARM include; “*I feel friendly toward my therapist*,” “*I feel accepted in therapy no matter what I say or do*,” “*I find therapy warm and friendly*,” and “*My therapist is supportive*.” These statements incorporate emotional language and ask the client to report on their feelings toward the therapist on an individual level. The other core dimensions of alliance measured by the ARM, partnership and confidence, focus more on practical issues and ask the client to comment on the working relationship such as “*My therapist and I agree how to work together*,” “*My therapist and I are willing to work hard together*,” “*I have confidence in the therapy and the techniques being used*,” and “*The professional skills of the therapist are impressive*” (11).

#### The impact of cannabis on clients’ perceptions of Bond

Client perspectives of therapeutic bond were significantly lower for current cannabis users than non-current users however this difference was not seen for the “Cannabis Clinic” groups. Given that the Bond dimension incorporates the emotional attachment between the client and clinician, one possible explanation is that the effects of current cannabis use might impact on a person’s ability to form this emotional connection. Perhaps a combination of the lower interpersonal sensitivity, heightened paranoia, loss of personal identity, and anxiety that have been associated with chronic cannabis use (15, 16, 18) result in the client feeling emotionally numbed or disconnected from the clinician during treatment. Anecdotally, current cannabis users often describe a pattern of smoking cannabis throughout the night and first thing when they wake of a morning. It may be that current cannabis users are likely to attend treatment whilst intoxicated by the effects of their drug then people who are seeking treatment for methamphetamine or alcohol who may have a different pattern of use. This may explain the reported differences and difficulties in establishing a therapeutic bond with their therapist.

### Exploring the impact of a specialized cannabis service on perceptions of alliance

Interestingly, people referred to the study from the Specialist Cannabis Clinic did not report a lower perception of therapeutic bond than clients seeking treatment from other teams. Perhaps this might be explained by treatment motivation. Cannabis Clinic clients typically want to engage in treatment for the purpose of reducing or ceasing their cannabis use. Perhaps, in this context, the partnership with the therapist around tasks and goals is established more easily, allowing the clinician more time to focus on their emotional connection with the client. This is perhaps one demonstration of the benefits and importance of a specialized cannabis treatment team, physically located separately from people seeking treatment for other drugs, and staffed by specialist clinicians with additional training and support in how to engage and manage people with current cannabis use problems.

Therapist-related factors may also have impacted on this result, as different clinicians were operating across the three clinical settings targeted in the current study. Given the development of therapeutic bond is one involving personal factors, and that the therapist too, brings their own qualities to the therapeutic relationship, it is also possible that these qualities differed across the clinics and thus contributed to the differences in results between clinics. A testament to this was the significantly lower ratings of the clinicians’ perspective of Bond, which was significantly lower for Cannabis Clinic clients than for clients from other teams. There was also a trend for clinicians from the Cannabis Clinic to report lower scores on the Confidence dimension then clinicians from other teams, suggesting that Cannabis Clinic clinicians have less confidence and optimism in therapy and their techniques. When considering these results it is important to note that there were a small number of people using cannabis in the other teams and individual clinician differences may have influenced these outcomes. However, these results may also indicate that clinicians working within the Specialized Cannabis Clinic are more aware of the difficulties of engaging cannabis users and in some way account or compensate for the lack of emotional connection with their clients. These findings provide useful information for individuals providing supervision or training for therapists working with cannabis clients.

### The impact of age, gender, and mental health on therapeutic alliance

In general, there was a tendency for the youngest age group to score lower on the client therapeutic alliance subscales, particularly for Openness. Males also scored consistently lower on the therapeutic alliance measure than females. There were no significant differences found between age and gender on the therapeutic alliance dimensions, in contrast to previous research (14). Perhaps in the current study, the effects of drug use over-ride any potential age and gender differences in the establishment of therapeutic alliance; an issue that warrants further investigation.

There were no significant correlations found between depression, anxiety, and stress (DASS-21) scores, the dimensions on the client therapeutic alliance measure and amount of cannabis use. This finding is similar to Diamond et al. (13) who found no significant impact of mental health symptoms on early therapeutic alliance in their study with young cannabis users. There were also no significant differences found between current and non-current cannabis users on the DASS-21 scores. This may have been due to a ceiling effect, whereby our study participants scored much higher than population norms on the depression, anxiety, and stress subscales. If the notion of the intoxication and chronic effects of cannabis use resulting in a person becoming emotionally disconnected is correct, then perhaps cannabis might impact on a person’s own level of insight into their emotional health, given the negative correlation between amount of cannabis use and perceived Bond with their clinician.

### Exploring the use of computer-based interventions for cannabis users

In general, our hypotheses related to differences between cannabis users and users of other drugs in terms of openness to computer/Internet-delivered treatment modalities were not supported. However, current cannabis users tended to be less anxious than non-current users about the idea of using computers, scoring lower on the CARS. Conversely, the current cannabis group reported fewer positive thoughts about computers than did their counterparts on the CTS. Both of these results were not significant. This finding potentially also supports the above notion of cannabis users being emotionally disconnected from their experiences in general, rather than specifically about the use of computers/the Internet. In this context, however, the potential advantage of this finding is that cannabis users may be willing to at least try alternative modes of delivery of treatment.

A higher proportion of people from the current cannabis use groups reported previous exposure to Internet-delivered treatments; thought that Internet-delivered treatments could be useful managing their primary drug of concern; and were more willing to use Internet-delivered treatment when compared to non-current cannabis users. Although not significant, these results generally highlight the potential of the Internet in supporting treatments for cannabis users. This particular modality of treatment is of relevance because the Internet can increase the reach of therapy outside an individual treatment session, and can overcome logistical and emotional barriers to attending regular treatment appointments with a therapist. Such barriers might include basic issues of organization and functioning, such as getting to the treatment session in a clinic, through to reducing any anxiety or reluctance that might be associated with interacting with a clinician in a treatment program. Internet-delivered treatment could also be used as a transition into therapy for current cannabis using clients by introducing psychological concepts and building insight. Future research could consider these issues specifically for cannabis users relative to those using other drugs.

Previous research has indicated there is no negative impact of computer-based treatments on the therapeutic alliance when integrated with existing psychosocial treatments (30), so given cannabis users reported particular difficulty building a therapeutic bond with their therapist, have heightened paranoia and interpersonal sensitivity, Internet-based treatments may be one potential way to engage, transition, or retain this growing population in treatment.

### Limitations

A limitation of this study was the referral of client participants by clinicians. Due to client confidentiality within the service this method appeared the most ethical option however it did leave the door open for clinician bias. Clinicians may have unintentionally put their own bias on their referrals potentially referring a certain type of client and not referring others. In addition, even though clinicians did not know which of their clients had actually consented to participate in the study they were aware of who had been referred, potentially impacting on their ratings of therapeutic alliance. It may also be difficult to generalize these findings beyond an outpatient treatment setting, as cannabis users who do not access treatment may be different from those who do. However, it is likely that similar issues with emotional numbing and interpersonal difficulties would be evident in non-treatment seeking cannabis users, and may in fact be pivotal in their decisions not to seek treatment. Future research with general community samples might be important to pursue.

## Conclusion

Cannabis users can be reluctant to seek treatment and there are often high treatment “drop-out” rates associated with counseling interventions, which have proven effectiveness, and are often preferred (20). Given that service factors have the ability to influence engagement with clients in drug treatment (31) and from the evidence found in this study, it is clear that research needs to focus on the engagement and retention of this particularly difficult client group from a service perspective, and how to better support and train clinicians working with cannabis users. Our results suggest that a focus on developing and improving the therapeutic bond between client and therapist is an important starting point in this process. The implementation of Internet/computer-based interventions for the treatment of cannabis use and associated problems may take us one step closer to improving treatment outcomes. To date, no research has investigated the effectiveness of computerized treatments, integrated with standard psychosocial treatments, specifically with cannabis users in a real world setting.

## Trial Registration

Australian Clinical Trial Registration Number: ACTRN12611000382976

## Conflict of Interest Statement

The authors declare that the research was conducted in the absence of any commercial or financial relationships that could be construed as a potential conflict of interest.
